# Initial steps for integrating academic electronic health records into clinical curricula of physical and occupational therapy in the United States: a survey-based observational study

**DOI:** 10.3352/jeehp.2022.19.24

**Published:** 2022-09-02

**Authors:** Stephen Burrows, Lola Halperin, Eric Nemec, Wendy Romney

**Affiliations:** 1 Department of Healthcare Informatics & Administration, College of Health Professions, Sacred Heart University, Fairfield, CT, USA; 2 Department of Occupational Therapy, College of Health Professions, Sacred Heart University, Fairfield, CT, USA; 3 Department of Physician Assistant Studies, College of Health Professions, Sacred Heart University, Fairfield, CT, USA; 4 Department of Physical Therapy and Human Movement Science, College of Health Professions, Sacred Heart University, Fairfield, CT, USA; Hallym University, Korea

**Keywords:** Curriculum, Electronic health records, Occupational therapy, Physical therapy, Problem-based learning

## Abstract

Training programs must be designed to prepare physical and occupational therapy students to use electronic health records (EHRs) and interprofessional collaboration. This report aims to describe physical and occupational therapy students’ perceptions of integrating an academic EHR (AEHR) in their problem-based learning (PBL) curricula in the College of Health Professions, Sacred Heart University, Fairfield, Connecticut, the United States. A paper-based case approach to PBL was adapted by creating patient cases in an AEHR. Students were asked to complete chart reviews and review provider notes to enhance their learning. An online survey was conducted to determine their perceptions of using AEHR from May 2014 to August 2015. Eighty-five students completed the survey, and 88.1% felt that using an AEHR was needed, and 82.4% felt that the additional notes enhanced their understanding of the interdisciplinary team. However, 83.5% reported the AEHR system increased the time needed to extract meaningful information. Incorporating an AEHR into curricula is essential to ensure students are adequately prepared for future patient interactions.

## Background/rationale

Over the past decade, the use of electronic health records (EHRs) has grown substantially. The Health Information Technology for Economic and Clinical Health (HITECH) Act of 2009 recognized EHRs as critical in ensuring the highest quality of patient care and safety [1]. Given the essential role EHRs play in patient care, incorporating them into curricula ensures students are prepared to enter contemporary practice and satisfy accreditation standards [2,3]. Some programs employ academic EHRs (AEHRs), modified versions of EHRs used in acute care and ambulatory facilities, for the needs of academic settings. The AEHR simulates a learning environment where students can interact with the functionality of EHRs while learning discipline-specific content and processes used in various clinical settings [4].

Employing simulated patient cases in an AEHR exposes students to the value of technology and its impact on healthcare and improves their confidence and critical thinking skills [5]. This interactive approach to learning the technology includes viewing patients’ clinical documentation, test results (laboratory tests, diagnostic imaging, etc.), performing chart reviews, medication administration, and developing care plans. Through these learning activities, the student begins to use evidence-based clinical practices, critical-thinking skills, and data-driven decision-making. Scarce literature has been published about integrating AEHR into didactic rehabilitation curricula, specifically problem-based learning (PBL). Still, the current findings emphasize the importance of further steps in this direction [6,7].

PBL is a student-centered approach to education. Its effectiveness in healthcare education is well-documented, including applications to interprofessional learning [5]. In PBL, students meet in small groups of 5–8 with a faculty facilitator. They are provided with patient cases that introduce them to diagnoses and symptomatology, common medical treatments, pharmacology, and discipline-specific assessments and interventions, as well as cultural and inter-professional collaboration aspects of healthcare provision and reception. Students identify unknown information, create a list of learning issues, and divide them among themselves, and then research and develop presentations on their respective topics to present to each other at the next session. The case studies drive the large-group discussions (lectures) and lab activities that follow.

## Objectives

This case report aims to describe physical and occupational therapy students’ perceptions of integrating an AEHR system into PBL curricula in the College of Health Professions, Sacred Heart University, Fairfield, Connecticut, the United States. Specifically, we would like to determine if the use of an AEHR provided a learning experience that improved student learning and to solicit feedback from the pilot offering.

## Ethics statement

This study was approved by Sacred Heart University’s Institutional Review Board (#140807A). The study survey was anonymous; therefore, the requirement to obtain informed consent was exempted by the Institutional Review Board.

## Study design

This was a survey-based observational study. The manuscript described the study according to the STROBE statement (https://www.strobe-statement.org).

## Setting

**Fig. 1.**
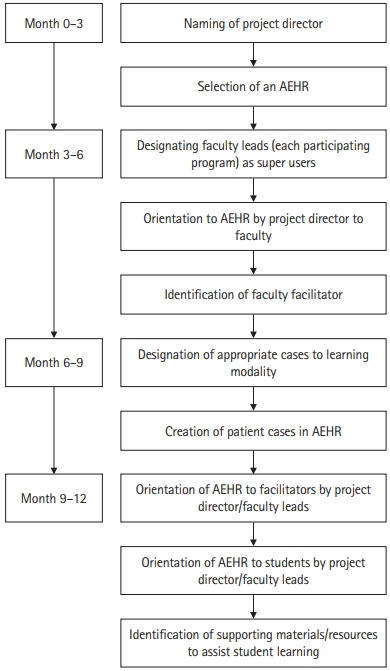
Process of implementing an academic electronic health record (AEHR) system into physical and occupational therapy curricula in the College of Health Professions, Sacred Heart University, Fairfield, Connecticut, the United States.

**Fig. 2.**
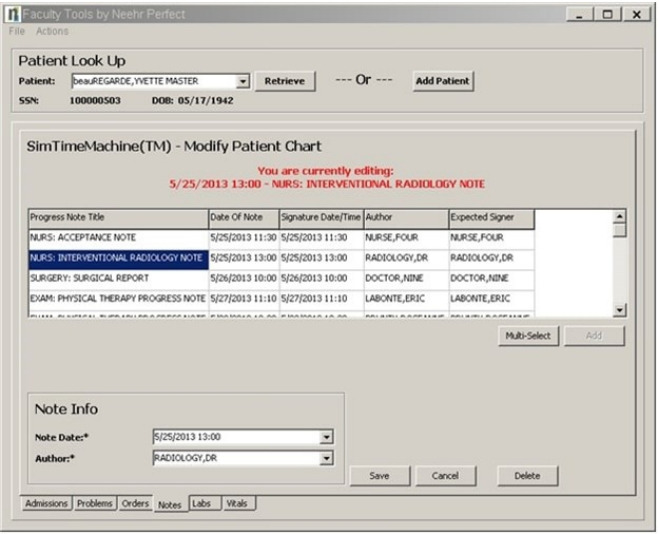
Example of the academic electronic health record: notes of patient/case Yvette Beauregarde.

Sacred Heart University students enrolled in the Doctor of Physical Therapy (PT) and Master of Occupational Therapy (OT) programs participated in a project using an AEHR system. Selection of the AEHR system was completed by the program director, who has a background in health information technology. The program director selected the AEHR system based on ease of use, customer training and support, ability to add cases, and cost. The process for integration is outlined in Fig. 1. Both programs utilize a PBL approach where patient cases drive the curriculum. PT students meet twice weekly for 6 hours to discuss, review, and present 26 patient cases each semester, for 5 didactic semesters. OT students meet once per week for a total of 3 hours to analyze 41 clinical cases over 3 semesters. Seven of the 125 paper cases were uploaded into the AEHR system for students in the PT program, and 7 out of the 41 paper cases were uploaded in the OT program (Supplement 1, Fig. 2).

This project was designed to increase students’ familiarity with EHR systems and enhance their ability to complete a chart review, extract valuable information from patient notes, and understand the roles and responsibilities of the interdisciplinary team. The AEHR system covers all settings (acute care hospital, rehabilitation, and outpatient). The selected cases include patients seen by physical or occupational therapists in the hospital, outpatient, and rehabilitation settings. The paper cases were converted to the AEHR system by creating health care provider notes, medication orders, and lab values. Health care provider notes may include physician, nursing, physical therapy, occupational therapy, group therapy notes, social work, case coordinator, radiology, and operative notes. In addition, laboratory values, medications, and orders for the patient were added to the patient cases for the students to navigate the system and find appropriate information.

Students completed one 75-minute lecture orienting them to the AEHR and a scavenger hunt to familiarize themselves with navigating a patient chart. Then they were provided with links to the patient cases to complete the chart audit. The faculty facilitator was provided with an instruction sheet guiding the students to review the chart and extract valuable information. A survey questionnaire was sent to the students anonymously via SurveyMonkey (Momentive, San Mateo, CA, USA) to determine their perceptions of using the AEHR system to augment the PBL curriculum from May 2014 to August 2015. Responses were collected from May 2014 to August 2015 (Supplement 2).

## Participants

In total, 176 PT and OT students were invited to participate within 6 months after interacting with the AEHR in the College of Health Professions, Sacred Heart University, Fairfield, Connecticut, the United States, from May 2014 to August 2105. These participants included 2 cohorts of PT students (cohort 1: n=68, 70.5% female, 97.1% 20–25 years old; cohort 2: n=63, 74.6% female, 95.2% 20–25 years old) and 1 cohort of OT students (n=45, 88.9% female, 93.0% 20–25 years old). There was no exclusion criterion.

## Variables

The variables were 7 items in the survey questionnaire about participants’ perceptions of using the AEHR.

## Data sources/measurement

An 11-item questionnaire was developed by the investigators (Supplement 2). It consisted of 2 binary, 2 open-ended, and seven 5-point Likert questions from “strongly disagree” to “strongly agree”. Those 7 items pertained to the use of AEHR systems, time demands, interdisciplinary notes, and learning experiences. Face validity was established by the investigators, who are experts in AEHR, PT, and OT. Content validation was established using a modified Delphi process. WR drafted the first version of the survey and sent it to SB and LH. One hundred percent consensus was achieved between the 3 investigators in the third round. The internal consistency reliability of the questionnaire was shown by a Cronbach’s α of 0.733. Raw response data from participants are available from Dataset 1.

## Bias

There was no bias in selecting participants because this study was based on voluntary participation.

## Study size

No study size estimation was done. Only voluntarily participating students were included in the survey.

## Statistical methods

Data were analyzed descriptively with numbers and percentages.

## Main results

Out of 176 target students, 85 (48.3%) consented and completed the survey: 71 PT students (83.5%) and 14 OT students (16.5%). Forty-six students (54.8%) had previous experiences of EHR use (Table 1). However, despite the familiarity with EHR navigation, most felt that the AEHR was more time-consuming than the traditional paper cases (83.5% selected either “strongly disagree,” “disagree,” or “neutral”). However, the majority felt that reviewing the additional provider notes enhanced their understanding of the interdisciplinary team (82.4% “agreed” or “strongly agreed”), which enhanced their learning (57.6% “agreed” or “strongly agreed”). While the AEHR was perceived as more time-consuming, respondents indicated value in this time investment. They recommended incorporating more AEHR use, as it was deemed necessary for rehabilitation provider education (88.1% “agreed” or “strongly agreed”). Fifty-one (60.0%) responded to the open-ended question that solicited suggestions for improvement. Thirty-four (40.0%) of these respondents indicated that they needed a more robust tutorial regarding AEHR functionality. Thirteen respondents indicated they had technical difficulties with the product; however, this appeared to primarily impact Mac users. While the prompt asked for suggestions, several respondents offered constructive reflections on the experience, stating that they now know that all EHRs function differently, and while this AEHR was unlike their previous experience, it prepared them to anticipate future challenges during fieldwork.

## Interpretation

We successfully integrated an AEHR into the PT and OT curricula to simulate the clinical environment. Paper cases were adapted to the AEHR, and new provider notes were added. Students believed the AEHR was necessary for education, and the additional provider notes enhanced their understanding of the interdisciplinary team. They found that the AEHR was more time-consuming than the traditional paper cases. The qualitative data also revealed frequent technical challenges. Faculty seeking to implement AEHR should anticipate the need for additional technology support during classroom training or consider tutorials in advance of the event to minimize technical difficulties during active learning seminars.

The nursing literature has also found the importance of using AEHR in curricula and the need for careful integration [8]. The time barrier was expected as students had to learn how to navigate the system, and new provider notes were added. Our findings resemble those reported by other healthcare practitioners. More specifically, increased time demands imposed by EHR utilization have been commonly cited as a barrier to use [8].

The authors believe integrating AEHR in the curricula is the initial step in covering the standards and required elements of the use of EHR for accreditation in PT and OT programs [3,4]. These standards include the Commission on Accreditation in Physical Therapy Education criteria 7D40 (use of health informatics in a health care environment) and the Accreditation Council for Occupational Therapy Education B.4.15 standard (knowledge of the use of technology in practice). Exposure to AEHR and simulated experiences with chart audits prepare students for the healthcare environment. More work is needed to determine if the integration of AEHR is useful after clinical education experiences and graduation. In addition, more robust study designs are needed to help determine the best strategies for integration.

The integration of AEHR improved students’ understanding of the interdisciplinary team. The value of interprofessional education is well recognized for effective collaboration among future health care professionals [9]. The successful implementation of interprofessional education employing AEHR has also been reported [7]. The incorporation of the AEHR into curricula meets Competency 2 and competency 3 of the Interprofessional Education Collaborative Core Competencies using multi-disciplinary progress notes and other clinical information “to use the knowledge to one’s own role and those of other professionals to address needs of patients” and to foster communications between “professionals in health and other fields” to “support a team approach to the promotion and maintenance of health and the prevention and treatment of disease” [10]. Future work should further explore the usefulness of integrating AEHR and the interdisciplinary team.

## Limitations

Potential limitations of the study include the small cohort of physical and occupational therapy students from a single university who were taught with the pedagogical approach of PBL. This study lacked randomization and had a single-group observational post-design.

## Generalizability

These findings support the importance of integrating AEHR into professional education curricula to prepare students for EHR in the healthcare environment.

## Conclusion

Students’ perceptions of the use of AEHR in the curriculum were overall favorable and supported the hypothesis that its use enhanced their learning. Students believed it was essential but reported it was more time-consuming than traditional paper cases due to the technical proficiency required to navigate the AEHR.

## Figures and Tables

**Table 1.** Physical and occupational therapy students’ perceptions of using the AEHR system in the College of Health Professions, Sacred Heart University, Fairfield, Connecticut, the United States

	No. of responses (%)				
	Strongly disagree	Disagree	Neutral	Agree	Strongly agree
Retrieving clinically relevant information from the AEHR took less time as compared to retrieving clinically relevant information from paper-based cases (n=85)	12 (14.1)	49 (57.6)	10 (11.8)	14 (16.5)	0
The AEHR cases are more intellectually stimulating than paper-based cases (n=84)	0	26 (30.6)	24 (28.2)	32 (37.6)	3 (3.5)
Additional provider notes (social workers, nurses, physicians, etc.) in the AEHR enhanced my understanding of the roles in an interdisciplinary team (n=84)	0	6 (7.1)	9 (10.7)	61 (72.6)	8 (9.5)
The AEHR cases provided more information about the patient/client as compared to paper-based cases (n=84)	0	19 (22.6)	31 (36.9)	25 (29.8)	9 (10.7)
The AEHR clinical cases have enhanced my learning as a future health professional (n=84)	0	13 (15.5)	23 (27.4)	44 (52.4)	4 (4.8)
I recommend further incorporating the AEHR system into my program’s curriculum (n=83)	1 (1.2)	14 (16.7)	19 (22.6)	41 (48.8)	9 (10.7)
Learning about EHR systems is necessary in education of students in the health professions (PT, OT, SLP) (n=85)	0	3 (3.6)	7 (8.4)	44 (53.0)	29 (34.9)

AEHR, academic electronic health record; EHR, electronic health record; PT, physical therapy; OT, occupational therapy; SLP, speech and language pathology.

## Footnotes

**Data availability**
Data files are available from Harvard Dataverse: https://doi.org/10.7910/DVN/UVG1TU Dataset 1. Raw response data from participants.

## Supplementary materials

Supplementary files are available from Harvard Dataverse: https://doi.org/10.7910/DVN/UVG1TU

Supplement 1. Summary of 14 cases uploaded to the academic electronic health record system, the College of Health Professions, Sacred Heart University, Fairfield, Connecticut, the United States.

Supplement 2. Survey questionnaire used to measure students’ perceptions of using the academic electronic health record system, the College of Health Professions, Sacred Heart University, Fairfield, Connecticut, the United States.

Supplement 3. Audio recording of the abstract.
